# Diabetes mellitus among HIV patients on ART at Woldia comprehensive specialized hospital, Northeast Ethiopia

**DOI:** 10.1038/s41598-025-96076-6

**Published:** 2025-03-29

**Authors:** Alene Geteneh, Seada Muhammed, Selamyhun Tadesse, Aragaw Tesfaye, Solomon Rega, Sirak Biset, Mulugeta Kiros

**Affiliations:** 1 Department of Medical Laboratory Science, College of Health Sciences, Woldia University, Woldia, Ethiopia; 2 Department of Internal Medicine, College of Health Sciences, Woldia University, Woldia, Ethiopia; 3 Amhara Public Health Institute, Woldia, Ethiopia; 4 Department of Medical Microbiology, School of Biomedical and Laboratory Sciences, College of Medicine and Health Sciences, University of Gondar, Gondar, Ethiopia; 5 Department of Medical Laboratory Science, College of Health Sciences, Raya University, Maichew, Ethiopia

**Keywords:** Antiretroviral therapy, Diabetes mellitus, Ethiopia, HIV, Risk factors, Prognosis, Quality of life

## Abstract

Diabetes mellitus (DM) is an emerging comorbidity among people living with HIV receiving antiretroviral therapy (ART), potentially impacting treatment outcomes, including virologic failure. Identifying key determinants of DM in this population is crucial for improving patient care. A hospital-based cross-sectional study was conducted from January 01 to May 30, 2024 at Woldia Comprehensive Specialized Hospital, Ethiopia. A total of 253 HIV patients on ART for at least six months were randomly selected. Data were collected via structured questionnaires, clinical measurements, and medical record reviews. DM was diagnosed on the basis of a fasting blood glucose level ≥ 126 mg/dl or random plasma glucose level ≥ 200 mg/dl. Logistic regression models were employed to identify factors associated with DM, reporting adjusted odds ratios (AORs) with 95% confidence intervals (CIs). Statistical significance was set at *p* < 0.05. The prevalence of DM among the study participants was 9.9%. Compared with female patients, male patients had a significantly greater risk of developing DM (AOR = 4.29, 95% CI = 1.079–17.04). A family history of DM was associated with a nearly 11-fold increased risk (AOR = 10.65, 95% CI = 2.82–40.20). Overweight individuals (BMI > 25 kg/m²) had a nearly sixfold-fold greater risk of DM (AOR = 5.95, 95% CI = 1.56–22.65). Additionally, ART interruption and restarting of ART were significantly associated with increased DM risk (AOR = 6.11, 95% CI = 1.88–19.84). Approximately one in ten HIV patients on ART had DM. The significant factors included male sex, family history of DM, overweight status, and ART interruption. These findings highlight the need for routine metabolic screening, targeted interventions and continuous monitoring to mitigate DM risk and optimize HIV care.

## Background

The commencement of highly active antiretroviral therapy (HAART) has significantly improved human immunodeficiency virus (HIV) management, enabling patients to live longer by effectively suppressing viral replication, reducing opportunistic infections, and preventing disease progression. However, the increased life expectancy of people living with HIV has led to a growing burden of comorbidities such as diabetes mellitus. Studies indicate that over 60% of HIV-positive patients aged ≥ 50 years have at least one comorbid condition and that those living with HIV are at a greater risk of developing DM than HIV-negative patients are^1,2^. This increased risk of DM is multifactorial. ART-induced metabolic dysfunction, mitochondrial toxicity, and insulin resistance contribute to the pathogenesis of DM. Moreover, HIV lipodystrophy syndrome, characterized by altered fat distribution and insulin resistance, further exacerbates glucose metabolism abnormalities^3,4^.

In sub-Saharan Africa (SSA), where HIV remains a major public health concern, DM is highly prevalent among ART users. For example, the prevalence of DM among HIV patients on ART in South Africa was 12.3%, with regional estimates ranging from 2.85 to 14.9%^5^. This double burden of communicable and noncommunicable diseases presents significant healthcare challenges, straining the already resource-limited healthcare systems. Dolutegravir (DTG) has been used as a first- and second-line treatment in sub-Saharan Africa (SSA) because of its enhanced pharmacological efficacy and virologic suppression over other ARTs^6^. However, case reports have shown that this drug is correlated with hyperglycemia^7,8^. Additionally, the high rates of virologic failure (11–24%) and HIV drug resistance (71–90%) further complicate ART treatment outcomes^9^. Despite the increasing recognition of HIV-DM comorbidity^10^, its determinants remain underexplored in Ethiopia. Thus, we aimed to assess the prevalence and risk factors for DM among HIV patients on ART.

## Materials and methods

### Study design and setting

A descriptive cross-sectional study was conducted between January 01 and March 30, 2024 at Woldia Comprehensive Specialized Hospital (WCSH), which is located in Northeast Ethiopia. This hospital serves a population of more than two million people and provides comprehensive HIV care, including ART services, with more than 4,166 clients being followed up during the study period.

### Eligibility criteria

The study included all HIV patients on ART for at least six months at WCSH. A total of 253 randomly selected HIV patients were recruited. The sample size determination was based on a previous study from Dessie Referral Hospital (DM prevalence = 8.8%)^11^, which used a 95% confidence interval. HIV-positive pregnant women and HIV-positive patients with preexisting diabetes or other comorbid conditions, including hypertension, cardiovascular diseases, chronic kidney disease, and tuberculosis were excluded from the study.

### Data collection, quality control, and measurements

Data were collected via a structured checklist that included both self-reported information and clinical assessments. Anthropometric measurements, including weight and BMI, were obtained via a calibrated digital weight scale. The blood glucose level was measured via a glucometer, and DM was diagnosed on the basis of a fasting blood glucose level ≥ 126 mg/dl or random plasma glucose ≥ 200 mg/dl^12^. Viral load test results were extracted from the medical record chart, and virologic failure was defined as plasma HIV RNA > 1000 copies/mL at the most recent visit^9^. The structured checklist included socio-demographic and clinical characteristics, including age, sex, educational status, marital status, residence, occupation, duration of ART, ART regimen type, baseline WHO HIV stage, family history of DM, BMI classification, history of ART interruption and re-initiation, missed clinic appointments, and viral load level. To ensure data accuracy and reliability, the questionnaire was pretested before actual data collection, and necessary modifications were made on the basis of the findings.

### Statistical analysis

The data were analyzed via SPSS version 25 (IBM, Chicago, IL). Both bi-variable and multivariable logistic regression models were used to identify factors associated with DM. Variables with a p value of < 0.3 in the binary analysis were included in the multivariable model. Statistical significance was set at a p value < 0.05, with the 95% CI reported for AOR.

### Ethical approval

Ethical approval was obtained from the Ethical Review Board of Woldia University. The concept of the study was explained to the study participants, written informed consent was secured from all the study participants, and confidentiality was strictly maintained. The study adhered to the Helsinki declaration on ethical research involving human subjects^13^.

## Results

### Socio-demographic and clinical characteristics of the respondents

A total of 253 participants were included in this study, with a mean age of 45.42 ± 10.75 years. More than 53% (*n* = 134) were aged ≥ 45 years. The majority were female (60.5%, *n* = 153), and 75.5% (*n* = 191) resided in urban areas. Regarding marital status, 49.8% (*n* = 126) were married. In terms of educational attainment, 26.1% (*n* = 66) had completed primary school.

Clinically, most participants (84.6%, *n* = 214) had been on ART for over five years, and 79.4% (*n* = 201) were on first-line ART regimens. A family history of DM was reported by 11.1% (*n* = 28) of the participants, and 31.17% (*n* = 79) were classified as overweight (BMI > 25 kg/m^2^) (Table 1).

**Table 1:** Socio-demographic, clinical, and behavioral characteristics of the respondents at Woldia comprehensive specialized hospital, 2024 (*N* = 253).

Variables	Category	Diabetes		Frequency	Percentage
		Yes	No		
Age in years	< 45	14	105	119	47
	>= 45	11	123	134	53
Sex	Female	7	146	153	60.5
	Male	18	82	100	39.5
Residence	Rural	10	52	62	24.5
	Urban	15	176	191	75.5
Marital status	Married	15	111	126	49.8
	Unmarried (single + divorced + widowed)	10	117	127	50.2
Occupational status	Farmer	6	43	49	19.3
	Government employee	5	25	30	11.9
	Housewife	2	79	81	32.0
	Merchant	12	81	93	36.8
Duration of ART	< = 5 years	6	33	39	15.4
	> 5 years	20	194	214	84.6
ART regimen	First line	14	187	201	79.4
	Second line	11	41	52	20.6
Family history of DM	Yes	12	16	28	11.1
	No	13	212	225	88.9
Body mass index(BMI)	Normal	12	168	180	71.1
	Underweight (< 18.5 kg/m^2^)	4	28	32	12.6
	Overweight (> 25 kg/m^2^)	9	32	41	16.2
Baseline WHO HIV stage	1	5	66	71	28.1
	2	5	66	71	28.1
	3	13	79	92	36.4
	4	2	17	19	7.5
Interruption and restarting of ART	Yes	14	40	54	21.3
	No	11	188	199	78.7
Missed clinic appointment	Yes	3	20	23	9.1
	No	22	208	230	90.9

### Prevalence of diabetes mellitus and virologic failure

The prevalence of DM among HIV patients on ART was 9.9% (*n* = 25). Virologic failure (HIV RNA ≥ 1000 copies/mL) was observed in 4% (*n* = 10) of the study participants. Notably, all six patients on second-line ART received DTG-based regimens (Table 2).

**Table 2:** Prevalence and simple correlation of diabetes and virologic failure among HIV patients on ART at Woldia comprehensive specialized hospital, 2024 (*N* = 253).

Prevalence	Frequency	Percentage	95% Confidence Interval
Diabetes Mellitus	25	9.9	6.3–13.4
Virologic Failure	10	4.0	1.6–6.3
	Type of ART Regimen		
	First	Second	Pearson Chi-Square = 9.921
	4	6	Sig = 0.002
	197	46	

### Factors associated with diabetes mellitus

Factors associated with the development of DM in the cohort were identified through bivariable and multivariable logistic regression analysis. The bivariable logistic regression analysis revealed that variables such as sex, residence, occupation, duration of ART, type of ART regimen, family history of DM, BMI, and interruption and restarting of ART medication had p values < 0.3; thus, these variables were included in the multivariable analysis. After multivariable logistic regression was performed to avoid possible confounding factors, only being male, having a family history of diabetes, being overweight, and being interrupted and restarting ART were found to be independently associated with an increased risk of diabetes.

Specifically, male patients on ART had a more than 4 times greater risk of developing DM than female patients on ART did (AOR = 4.29, 95% CI = 1.079–17.04). Additionally, having a family history of DM was a significant risk factor, with individuals being nearly 11 times more likely to develop DM than those without such a history (AOR = 10.65, 95% CI = 2.82–40.20). Interestingly, patients with a BMI above 25 kg/m²/overweight had a greater risk of developing DM than did those with a normal BMI (AOR = 5.95, 95% CI = 1.56–22.65). Other factors, interrupting and restarting ART medication, were also significantly associated with an increased risk of diabetes (Table 3).

**Table 3:** Bivariable and multivariable analysis for factors associated with diabetes mellitus among HIV patients on ART at Woldia comprehensive specialized hospital, Northeast Ethiopia, 2024 (*N* = 253).

Category	Diabetes		COR (95%CI)	*P* value	AOR (95%CI)	*P* value
	Yes, *n* (%)	No, *n* (%)				
Female	7	146	1		1	
Male	18	82	4.58(1.84; 11.42)*	0.001	4.29(1.08; 17.04)**	0.039
Rural	10	52	1		1	
Urban	15	176	0.44(0.19; 5.32)	0.063	0.35(0.94; 1.13)	0.12
Farmer	6	43	1		1	
Government employee	5	25	1.43(0.4; 5.18)	0.583	6.33(0.83; 48.35)	0.075
Housewife	2	79	0.18(0.035; 0.94)*	0.040	1.37(0.15; 12.43)	0.778
Merchant	12	81	1.06(0.37;3.03)	0.911	1.38(0.29; 6.55)	0.685
<=5years on ART	6	33	1		1	
> 5 years on ART	20	194	0.536(0.199, 1.44)	0.216	1.30(0.33, 5.07)	0.704
First line ART	14	188	1		1	
Second line ART	11	40	3.58(1.52; 8.46)*	0.004	1.59(0.50; 5.04)	0.430
Family history of DM	12	16	12.23(4.80; 31.15)*	0.0001	1065(2.82; 40.20)**	0.001
No family history of DM	13	212	1		1	
Normal	12	168	1		1	
Underweight	4	28	2.00(0.60, 6.64)	0.258	3.07(0.68, 13.82)	0.144
Overweight	9	32	3.94(1.53, 10.11)*	0.004	5.95(1.56, 22.65)**	0.009
Yes for ART interruption and restarting	14	40	5.98(2.53; 14.14)*	0.0001	6.11(1.88; 19.84)**	0.003
No for ART interruption and restarting	11	188	1		1	

## Discussion

This study revealed that 9.9% of HIV patients on ART had DM, a prevalence comparable to that reported by South Wollo (8.8%)^11^, Felege Hiwot Referral Hospital (FHRH) and Debre Markos Referral Hospital (8.8%)^14^, and global estimates from Zambia (5%)^15^, Brazil (7.14%)^16^, Malawi (5–13.2%)^17^, Senegal (14.5%)^18^, and Tanzania (17%)^19^. An increased risk of DM in HIV patients has been documented^20^, with mechanisms including chronic inflammation^21,22^and alterations in the gut microbiome^23^, which in turn are associated with metabolic abnormalities such as insulin resistance that lead to increased blood glucose levels^24^. In addition, ART regimens, especially protease inhibitors (PIs), such as lopinavir, are reportedly associated with the development of insulin resistance and increased inflammatory status, resulting in glucose metabolism disorders^25–27^. Furthermore, emerging evidence suggests that DTG-based ART regimens are associated with hyperglycemia^28,29^.

Our study revealed that a family history of DM, male sex, overweight status, and ART interruption were significant risk factors. HIV-positive patients with a positive family history were nearly 11 times more likely to develop DM, which is consistent with studies in Ethiopia (Dessie^11^and Debre Tabor^30^) and other international works^31–33^. Genetic predispositions that affect insulin regulation and glucose metabolism, as well as lifestyle factors shared with families that lead to unhealthy habits, are linked to an increased risk of developing diabetes^34^. Although patients have no control over unmodifiable factors such as genetic susceptibility, they can avoid the impact of modifiable factors such as unhealthy lifestyle behavior, including unhealthy diet and physical inactivity, on the development of DM^35^.

Increased body fat is a well-known contributor to insulin resistance, which explains why overweight (BMI > 25 kg/m²) participants have a sixfold greater risk of DM^36–40^. Previous studies in Zambia and Brazil also identified sex, overweight status, and family history of DM as risk factors associated with DM in HIV patients^15,16^. Although a longer duration of ART use is associated with a higher prevalence of DM, this was not the case in our study. Despite our findings, studies in Dessie^41^, Jimma^42,43^, and southern Ethiopia^44^ indicate that long-term ART use is associated with an increased risk of metabolic disorders.

Males had a significantly greater risk of DM, which aligns with studies in Zambia and Brazil^15,16^. Differences in fat distribution, hormonal profiles, and lifestyle factors may contribute to this disparity^45–47^. Patients with ART interruption had a greater than 6-fold greater risk of DM, highlighting the need for consistent ART adherence to minimize metabolic complications. Interruptions may exacerbate metabolic complications by destabilizing the viral load, increasing the risk of insulin resistance and subsequent diabetes. Although the underlying reasons for the association between diabetes and virologic failure warrant further investigation, 4% of the participants experienced virologic failure in the current study. One study reported the importance of ART adherence and controlled DM among HIV patients in decreasing HIV viremia. According to this study, HIV-positive women with DM who are highly adherent to ART have a lower risk of viremia than do those with poor ART adherence^48^.

### Study limitations

Despite its valuable findings, this study has several limitations, including its cross-sectional study design, limited sample size, and, owing to resource unavailability, DM was merely diagnosed on the basis of fasting and random blood glucose levels. The sensitivity and specificity of random blood sugar and fasting blood sugar tests for diagnosing DM are lower than those of an oral glucose tolerance test (OGTT), the gold standard test for diagnosing DM and pre-diabetes^49,50^. This may underestimate the prevalence of DM in our study.

## Conclusions and recommendations

This study revealed that 9.9% of HIV patients on ART had DM, with significant risk factors, including male sex, family history of DM, overweight status, and ART interruption. While virologic failure was observed in 4% of the participants, further research is needed to explore the potential links among DM, ART regimens, and virologic suppression. We recommend routine metabolic screening for HIV patients on ART, especially those with risk factors for DM; lifestyle interventions such as weight management to prevent metabolic complications; continuous ART adherence monitoring to reduce the risk of both DM and virologic failure; and further longitudinal research to explore associations between DM, ART regimens, and virologic suppression.

## Data Availability

All the information used for analysis in the current study is available from the corresponding author and will be shared upon reasonable request.
